# Editorial: Integrating AI in forensic medicine: advancements and ethical considerations

**DOI:** 10.3389/fmed.2026.1873009

**Published:** 2026-06-22

**Authors:** Gianpietro Volonnino, Michele Treglia, Raffaele La Russa

**Affiliations:** 1 Department of Medicine, UniCamillus-Saint Camillus International University of Health Sciences, Rome, Italy; 2 Department of Biomedicine and Prevention, University of Tor Vergata, Rome, Italy; 3 Department of Clinical Medicine, Public Health, Life Sciences, and Environmental Sciences, University of L'Aquila, L'Aquila, Italy

**Keywords:** artificial intelligence, deep learning, forensic imaging, forensic medicine, forensic psychiatry, metabolomics

Forensic medicine is a highly versatile and multifaceted discipline that spans medicine, law, forensic pathology, forensic psychiatry, and bioethics. The development and exponential growth of artificial intelligence systems have naturally also impacted the field of forensic medicine. This editorial aimed to analyze both the potential benefits and the inherent risks associated with the application of artificial intelligence across the various branches of forensic medicine.

The article by Orsini et al., following a systematic review and analysis of 18 studies published between 1990 and 2025, examined the areas of application of AI in forensic medicine. The results of this study showed that AI has multiple applications. First, it enables the analysis of gunshot wounds, allowing the identification of shooting distance and the distinction between entry and exit wounds. At the same time, the study highlighted how post-mortem CT can be highly useful in determining the cause of death. Moreover, deep learning models have proven effective in accurately identifying diatoms for the diagnosis of drowning.

Another application concerns forensic odontology. AI can, in fact, estimate dental age with very high precision.

Last but not least, AI has applications in forensic psychiatry, where it has been shown to support the assessment of mental illness.

The study therefore concludes that AI should be considered a decision-support tool, while the final decision must remain human, especially in light of its ethical and legal implications.

The second published article, by Chen et al., focused on the study of the post-mortem interval (PMI) using AI-based methods. This is because traditional methods (cadaveric phenomena, entomology, microbiology) are often unreliable and lack objectivity. Therefore, the authors proposed analyzing variations in skeletal muscle metabolites.

Specifically, the authors analyzed femoral muscle samples taken from 32 rats and maintained at 35 °C. For the analysis of these samples, they used a metabolomics technique based on UPLC/Q-TOF mass spectrometry, which allows the identification and quantification of hundreds of metabolic molecules present in tissues. The study identified 14 significantly altered metabolites, seven of which were proposed as potential PMI biomarkers (including L-threonine, L-tryptophan, N6-acetyl-lysine, creatine, and glycerol-3-phosphate). Variations in the concentration of these metabolites are associated with post-mortem autolysis and tissue degradation processes. In particular, the study highlighted alterations in two metabolic pathways: the arachidonic acid pathway and the tryptophan pathway. Thus, the study concludes that analyzing changes in the concentration of these metabolites may be useful for estimating the PMI.

The study by Delogu et al. aimed to analyze gunshot wounds and provide a specific classification using deep learning. The authors developed an artificial intelligence model based on convolutional neural networks that analyzed photographic images of gunshot wounds. The results demonstrated the usefulness of AI in recognizing entry and exit wounds and estimating shooting distance. The study concludes that AI could be a valuable tool for forensic experts; however, a larger number of images is needed to train the models adequately and allow their use in judicial settings.

The study by Wang et al. presents a machine learning system called BIPON (Biomechanical Informed Predictive Optimization Network), capable of integrating imaging data, temporal information, and biomechanical parameters to improve the interpretation of diagnostic evidence. The authors applied the model particularly to the analysis of knee MRI images. The results suggest that the BIPON system is effective in improving both discrimination and robustness in imaging-based analyses.

The challenge in the coming years will be to integrate these new artificial intelligence methodologies into daily forensic practice. Achieving this will require collaboration among forensic physicians, engineers, biostatisticians, and legal experts.

From this perspective, AI should never be considered a replacement for the forensic expert, but rather a complementary tool aimed at enhancing the analytical capabilities of the specialist. Forensic interpretation remains a complex process that requires not only technical expertise but also critical judgment, contextualization of data, and an understanding of the legal implications of scientific conclusions.

Ultimately, the evolution of artificial intelligence technologies offers forensic medicine the opportunity to enter a new phase of methodological development through automated data analysis. However, the true value of these tools will always lie in their ability to support—rather than replace—the scientific judgment and professional experience of the forensic expert.

